# Rare Inflammatory Myofibroblastic Tumor of the Urinary Bladder: A Case Report and Review of the Literature

**DOI:** 10.3390/jcm15052047

**Published:** 2026-03-07

**Authors:** Zilvinas Venclovas, Agne Talackaite, Gabija Dadurkaite, Stasys Auskalnis, Mindaugas Jievaltas, Ieva Rubaviciute, Daimantas Milonas

**Affiliations:** 1 Department of Urology, Lithuanian University of Health Sciences, LT-44307 Kaunas, Lithuania; stasys.auskalnis@lsmu.lt (S.A.); mindaugas.jievaltas@lsmu.lt (M.J.); daimantas.milonas@lsmu.lt (D.M.); 2 Faculty of Medicine, Medical Academy, Lithuanian University of Health Sciences, LT-44307 Kaunas, Lithuania; agne.talackaite@stud.lsmu.lt (A.T.); gabija.dadurkaite@stud.lsmu.lt (G.D.); 3 Department of Pathological Anatomy, Lithuanian University of Health Sciences, LT-44307 Kaunas, Lithuania; ieva.rubaviciute@lsmu.lt

**Keywords:** inflammatory myofibroblastic tumor, IMT, urinary bladder tumor, IMTUB, TURB

## Abstract

**Background**: An inflammatory myofibroblastic tumor (IMT) is a rare mesenchymal tumor, sometimes with urinary bladder involvement (though this is extremely uncommon). Due to its rarity, the exact etiology and optimal treatment strategy remain unclear. **Methods**: A review of the existing literature on IMT of the urinary bladder was performed. **Results**: We report a case of a 32-year-old female presenting with frequent urination, hematuria with clots, and lower abdominal pain for one month. Initially misdiagnosed as acute cystitis, the symptoms persisted despite antibiotic therapy. Laboratory findings revealed severe anemia, and imaging studies identified a large bladder mass. Transurethral resection of the bladder tumor (TURB) was performed, and a 96 g mass was removed. Histopathological examination confirmed IMT of the urinary bladder (IMTUB) with positive immunohistochemical staining for ALK, vimentin, and actin. Follow-up at 30 months showed no recurrence, with annual cystoscopy and CT scans confirming remission. **Conclusions**: IMTUB should be considered in young patients presenting with hematuria and lower urinary tract symptoms. Early diagnosis through cystoscopy, imaging, and histopathological confirmation is essential for appropriate management. TURB remains the gold standard for treatment, with ALK inhibitors providing additional therapeutic options in select cases. Long-term follow-up is necessary due to the unknown malignant potential of IMTUB.

## 1. Introduction

An inflammatory myofibroblastic tumor (IMT) is a rare soft-tissue mesenchymal tumor occurring mainly in the abdominal cavity, retroperitoneum, pelvis, lung, and head and neck [1,2,3]. Approximately 75% of cases originate within the abdominal cavity, including the greater omentum, mesentery, and retroperitoneal space [4]. ITM affects both children and young adults, with a reported mean age at diagnosis of 38 years [5,6]. According to the National Cancer Institute, IMT is extremely uncommon, occurring in fewer than one per million individuals [7].

The etiology of IMT remains unclear, although associations have been reported with autoimmune system disease, smoking, Human Herpesvirus-8 and Epstein–Barr virus [8]. Involvement of the genitourinary system is particularly rare, accounting for less than 1% of all bladder tumors [9]. Generally, symptoms of IMT depend on the localization and size of the tumor [7]. The typical symptoms of an inflammatory myofibroblastic tumor of the urinary bladder (IMTUB) include hematuria, dysuria, frequent urination and pain in the lower part of the abdomen [6].

IMT is often considered a pseudotumor because it lacks marked cellular atypia and atypical mitotic activity. Histologically, the tumor is composed of spindle-shaped myofibroblasts accompanied by an inflammatory infiltrate consisting of eosinophils, plasma cells and lymphocytes [10,11]. Accurate differentiation from sarcomatoid carcinoma and leiomyosarcoma is essential to ensure appropriate treatment [12]. To date, there is no clear consensus regarding optimal management and follow-up strategies for patients with IMTUB [13].

In this study, we present a clinical case of a young woman diagnosed with a very rare IMTUB.

## 2. Materials and Methods

A focused literature review was conducted to identify reported cases of inflammatory myofibroblastic tumor (IMT) of the urinary bladder. A comprehensive bibliographic search was performed using PubMed, ScienceDirect, and other relevant databases, including manual screening of the reference lists of selected articles. The search included studies published up to December 2024 and was limited to articles written in English.

The search strategy used combinations of the following keywords: inflammatory myofibroblastic tumor, IMT, urinary bladder tumor, IMTUB, and TURB. Eligible publications consisted of case reports describing IMT of the urinary bladder. Exclusion criteria included studies on non-inflammatory myofibroblastic tumors, tumors located outside the urinary bladder, and non-English publications.

Articles were identified through searches of PubMed and ScienceDirect databases, and relevant references were screened manually. Duplicate records were removed prior to screening.

Data extracted from the included studies comprised patient demographics, clinical symptoms, diagnostic methods, treatment strategies, clinical outcomes, and prognosis.

Since the present study represents a clinical case report with a literature review, no chemicals, reagents, laboratory instruments, devices, or commercial cell lines/materials were used.

## 3. Case Report

**Figure 1:**
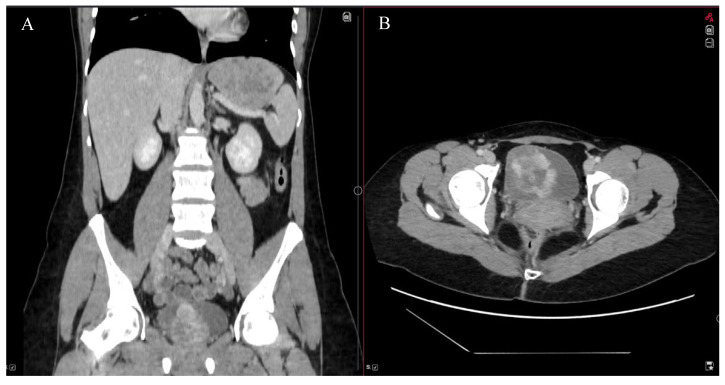
Frontal (**A**) and axial (**B**) CT scan of abdomen and pelvis showed a 5.3 × 5.1 × 5.8 cm mass in the urinary bladder, which accumulated contrast material.

A 32-year-old female suffered from frequent urination, hematuria with clots, and pain in the lower part of the abdomen. These symptoms lasted for 1 month. Acute cystitis was suspected. A single dose of 3 g Fosfomycin and non-steroidal anti-inflammatory drugs was prescribed. However, due to the remaining symptoms, nitrofurantoin was given. Prescribed medications did not lead to any improvement either. A general blood test was performed and it indicated severe anemia (Hb 46 g/L) without any signs of inflammation. Urinalysis revealed hematuria, while the urine culture test showed no presence of bacteria. Ultrasound demonstrated a partially filled bladder with heterogeneous masses (6.3 × 5.2 × 4.8 cm) with registered active blood flow. During the cystoscopy, a huge mass and bleeding were observed on the right side of the bladder, above the right ostium of the ureter. The abdominal and pelvic computed tomography (CT) scan identified a 5.3 × 5.1 × 5.8 cm mass that was accumulating contrast material (Figure 1). The right wall of the urinary bladder was deformed. Magnetic resonance imaging (MRI) was requested to evaluate the extent of the mass’s involvement in the layers of the bladder wall, but it was not performed due to claustrophobia.

Erythrocyte mass transfusion was performed due to severe anemia. Transurethral resection of the bladder (TURB) was performed. During surgery, a large bleeding tumor in the right-sided wall of the bladder, beginning above the right ureteral orifice, was identified. Tumor resection was performed. A total of 96 g of mass was removed from the urinary bladder. The patient was discharged without any symptoms five days after the operation.

**Figure 2:**
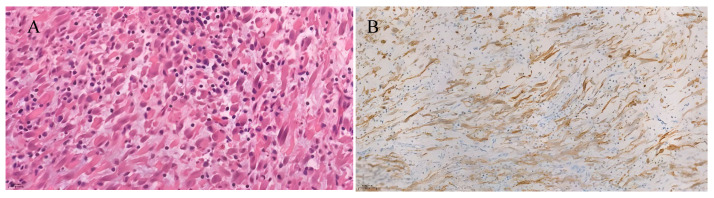
Immunohistopathologic findings of bladder tumor. (**A**) Hematoxylin and eosin (H&E) stain shows spindle cells in a background of transitional epithelial lining. (**B**) ALK expression.

The pathological–histological examination showed a tumor with medium cellularity, which was composed of fibroblast/myofibroblast-like cells arranged in fibrous structures with moderately abundant polymorphous inflammatory infiltration composed of eosinophils, plasma cells and lymphocytes (Figure 2). Tumor cells had positive reactions with vimentin, actin, ALK, focally with CK18, EMA immunomarkers. Reactions with CK7, GATA3, p63, and MyoD1 were negative. On the surface of some fragments, reactive urothelium with positive reactions of CK7 and GATA3 immunomarkers was identified. An inflammatory myofibroblastic tumor of the urinary bladder was diagnosed.

After 3 months, the patient was hospitalized for repeated TURB. The patient had a 4 cm scar after the previous operation and no clear signs of disease recurrence. Resection of the scar was performed for pathological–histological examination. The pathologist concluded that urothelium did not show atypia and no recurrence was seen. A CT scan was performed after 6 and there was no disease recurrence. Cystoscopy has been done annually, without any sign of disease recurrence. The follow-up period of this patient is 30 months.

## 4. Discussion

### 4.1. Epidemiology and Etiology

IMT of the urinary bladder is exceptionally uncommon [14]. Cases predominate in young females, comprising approximately 51.9% of reported patients (Supplementary Table S1) [15]. Because IMTUB is rare, its etiology remains incompletely understood, and the limited number of documented cases in the literature results in the absence of further treatment decisions [7,8]. A review of the literature was performed to better understand the clinical presentation, diagnostic challenges, and management strategies of IMTUB and follow-up methods.

### 4.2. Clinical Presentation

According to Jeremy Yuen Chun et al., the most common symptoms of IMTUB are hematuria (71.9%), dysuria (19.8%), frequent urination (18.8%) and lower abdominal pain (13.5%) [6]. In cases of bleeding tumor, anemia can occur due to hematuria [16]. However, these signs are non-specific and this is why it is difficult to diagnose IMTUB [17,18]. In our presented case, the patient had all of these symptoms, but were misdiagnosed with cystitis.

### 4.3. Diagnostic Evaluation and Histopathological Features

Cystoscopy allows us to specify the localization of IMT in the urinary bladder and CT scan helps us to identify changes in the abdominal and pelvic regions. However, an IMTUB diagnosis is mainly based on an immunohistochemical examination of the postoperative material [14]. The histological structure of the tumor consists of proliferated myofibroblasts with inflammatory infiltrates—lymphocytes, plasma cells, eosinophils, and histiocytes [15,19]. Immunohistochemistry describes positive reactions of tumor cells with vimentin, cytokeratin, and ALK, and expression of these genes plays a significant role in the biology of IMT and has been reported in about 65% of cases [20,21]. In our clinical case, tumor cells had positive reactions with these immunomarkers.

### 4.4. Differential Diagnosis

It is important to distinguish IMT from sarcomatoid carcinoma and leiomyosarcoma. Atypical mitoses, hyperchromasia, and cellular pleomorphism help to differentiate IMT from leiomyosarcoma, owing to the fact that these features should be expressed in leiomyosarcoma but not in IMT. In the case of sarcomatoid carcinoma, the p63 marker is found. However, IMT does not have this specific marker [12].

### 4.5. Treatment Methods

The main treatment for IMTUB is surgery—TURB. The procedure is performed to relieve symptoms, lower the risk of anemia which can be caused by bleeding tumors, and prevent the tumor from penetrating the muscular layer of the urinary bladder [11,22]. This tumor cannot be left for monitoring; it must be removed owing to its rarity and unknown likelihood of metastasizing. However, IMTUB is typically locally aggressive and only one case showed distant metastasis to the peritoneum and large intestine [23,24]. Radiotherapy has been successful in a few IMT cases, at doses of 20–40 Gy. Unfortunately, there are no reported cases in which radiation therapy has been used as the main treatment for IMTUB [25,26]. In our clinical report, it was decided to perform the main treatment of IMTUB and the large bleeding tumor was removed via TURB.

In September of 2022, the US Food and Drug Administration approved an ALK inhibitor, crizotinib, for adults and pediatric patients who have a positive reaction to ALK and whose tumors are inoperable or recurrent [14]. Inoperable tumors grow in locations within the body that are inaccessible to surgery; in these situations, attempting to operate could potentially damage vital tissues. Metastatic tumors are also considered inoperable [27].

According to Sophie Reinhart et al. [3], in their report on a clinical case described in 2020, if the tumor is inoperable and cannot be treated surgically, lorlatinib, a new-generation ALK inhibitor drug, can be used for IMTUB treatment. In their case, the physicians decided to reduce the tumor volume with the first-generation ALK inhibitor crizotinib and perform organ-sparing resection. However, no response was observed after 2 and 4 months of treatment. They decided to switch the medication to the next-generation ALK inhibitor lorlatinib and, in 5 weeks, an instant response with partial remission was identified and the sparing operative approach could be implemented. Based on this information, IMTUB, which has an ALK-positive reaction, can be successfully treated with the newer agent lorlatinib [3]. However, this treatment strategy was not applicable in our case because our patient’s tumor was operable and surgical management was feasible. Therefore, the patient underwent surgical treatment (TURB), and targeted therapy with ALK inhibitors was not required.

### 4.6. Follow-Up Prognosis

According to Jeremy Yuen Chun Teoh et al., the local IMT recurrence rate is only 4% [6]. However, it is still important to follow up patients with IMTUB due to its unknown malignant potential. Currently, there are no established postoperative surveillance protocols for IMTUB. Follow-up usually includes cystoscopy or CT scans performed every 3–6 months during the first year after surgery. After the first two years, cystoscopies can be repeated once every six months. Although the ideal follow-up duration is not well established, we recommend monitoring this tumor for at least five years due to its uncertain malignant potential [28].

## 5. Conclusions

IMT is a rare soft-tissue neoplasm characterized by infiltration of inflammatory cells. Involvement of the urinary bladder is extremely uncommon. Typical symptoms include lower abdominal pain, dysuria, and hematuria. Diagnosis is based on cystoscopy and CT imaging, with confirmation by postoperative immunohistochemical analysis. TURBT is the treatment of choice, and IMT of the urinary bladder generally follows a benign course with a favorable prognosis.

## Data Availability

Due to the case-report nature of this methodology, the data presented in this study are available on request from the corresponding author. No new data were created or analyzed in this study.

## Supplementary Materials

The following supporting information can be downloaded at https://www.mdpi.com/article/10.3390/jcm15052047/s1, Table S1. Summary of Prior Case Reports of Inflammatory Myofibroblastic Tumor (IMT) of the Bladder.

## References

[1] Marais B., Eyal P., Kesner K., John J. (2022). Inflammatory myofibroblastic tumour of the bladder: A case report and review of the literature. Ther. Adv. Urol..

[2] Derimachkovski G., Georgiev G., Sancha F.G., Danailova P., Yanev K. (2023). Rare inflammatory myofibroblastic tumor of the urinary bladder. Urol. Case Rep..

[3] Reinhart S., Trachsel Y., Fritz C., Wagner U., Bode-Lesniewska B., John H., Pless M. (2020). Inflammatory Myofibroblastic Tumor of the Bladder with FN1-ALK Gene Fusion: Different Response to ALK Inhibition. Urology.

[4] Siemion K., Reszec-Gielazyn J., Kisluk J., Roszkowiak L., Zak J., Korzynska A. (2022). What do we know about inflammatory myofibroblastic tumors?—A systematic review. Adv. Med. Sci..

[5] Gleason B.C., Hornick J.L. (2008). Inflammatory myofibroblastic tumours: Where are we now?. J. Clin. Pathol..

[6] Teoh J.Y.C., Chan N.H., Cheung H.Y., Hou S.S.M., Ng C.F. (2014). Inflammatory myofibroblastic tumors of the urinary bladder: A systematic review. Urology.

[7] Inflammatory Myofibroblastic Tumor-NCI. https://www.cancer.gov/pediatric-adult-rare-tumor/rare-tumors/rare-soft-tissue-tumors/inflammatory-myofibroblastic-tumor.

[8] Gros L., Dei Tos A.P., Jones R.L., Digklia A. (2022). Inflammatory Myofibroblastic Tumour: State of the Art. Cancers.

[9] Xu H., He B., Tu X., Bao Y., Yang L., Zhuo H., Wei Q. (2018). Minimally invasive surgery for inflammatory myofibroblastic tumor of the urinary bladder: Three case reports. Medicine.

[10] Zhu L., Li J., Liu C., Ding W., Lin F., Guo C., Liu L. (2017). Pulmonary inflammatory myofibroblastic tumor versus IgG4-related inflammatory pseudotumor: Differential diagnosis based on a case series. J. Thorac. Dis..

[11] Chen C., Huang M., He H., Wu S., Liu M., He J., Zang H., Xu R. (2022). Inflammatory Myofibroblastic Tumor of the Urinary Bladder: An 11-Year Retrospective Study from a Single Center. Front. Med..

[12] Alderman M., Kunju L.P. (2014). Inflammatory Myofibroblastic Tumor of the Bladder. Arch. Pathol. Lab. Med..

[13] EMA Recommends Extension of Indications for Crizotinib. https://www.esmo.org/oncology-news/ema-recommends-extension-of-indications-for-crizotinib.

[14] Song D., Jiao W., Gao Z., Liu N., Zhang S., Zong Y., Fang Z., Fan Y. (2019). Inflammatory myofibroblastic tumor of urinary bladder with severe hematuria: A Case report and literature review. Medicine.

[15] Inamdar A.A., Pulinthanathu R. (2019). Malignant transformation of inflammatory myofibroblastic tumor of urinary bladder: A rare case scenario. Bladder.

[16] Collin M., Charles A., Barker A., Khosa J., Samnakay N. (2015). Inflammatory myofibroblastic tumour of the bladder in children: A review. J. Pediatr. Urol..

[17] Li Y.P., Han W.W., Yang Y., He L.J., Zhang W.P. (2020). Inflammatory Myofibroblastic Tumor of the Urinary Bladder and Ureter in Children: Experience of a Tertiary Referral Center. Urology.

[18] Balagobi B., Gobishangar S., Ginige A., Gamlaksha D., Sanjeyan J., Suvethini L. (2022). Inflammatory myofibroblastic tumour: Case report of a rare form of bladder tumour. Int. J. Surg. Case Rep..

[19] Eshraghi B., Sonbolestan S.A., Abtahi M.A., Mirmohammadsadeghi A. (2019). Clinical characteristics, histopathology, and treatment outcomes in adult and pediatric patients with nonspecific orbital inflammation. J. Curr. Ophthalmol..

[20] Cessna M.H., Zhou H., Perkins S.L., Tripp S.R., Layfield L., Daines C., Coffin C.M. (2001). Are myogenin and myoD1 expression specific for rhabdomyosarcoma? A study of 150 cases, with emphasis on spindle cell mimics. Am. J. Surg. Pathol..

[21] Antonescu C.R., Suurmeijer A.J.H., Zhang L., Sung Y.-S.M., Jungbluth A.A., Travis W.D., Al-Ahmadie H., Fletcher C.D., Alaggio R. (2015). Molecular Characterization of Inflammatory Myofibroblastic Tumors with Frequent ALK and ROS1 Fusions and Rare Novel RET Gene Rearrangement. Am. J. Surg. Pathol..

[22] Wang C.S., Guu S.J., Wang C.J., Yang S.F., Ke H.L., Lee Y.C., Jhan J.H. (2021). A rare noncancerous but life-threatening tumor in urinary bladder. Clin. Case Rep..

[23] Matsui Y. (2021). A case of inflammatory myofibroblastic tumor of the urinary bladder with emergency clinical symptoms similar to bladder cancer. Urol. Case Rep..

[24] Libby E.K., Ellis L.T., Weinstein S., Hammer R.D., Murray K.S. (2019). Metastatic inflammatory myofibroblastic tumor of the bladder. Urol. Case Rep..

[25] Biswas R., Halder A., Gangopadhyay M., Biswas D. (2020). Inflammatory myofibroblastic tumor of maxillary sinus successfully treated with radiotherapy and corticosteroid: Report of a rare case. J. Egypt. Natl. Cancer Inst..

[26] Nkwam N., Johnson B., Bazo A., McCulloch T.A., Mann G.S. (2016). Inflammatory myofibroblastic tumour of the urinary bladder managed with partial cystectomy: A case report & literature review. J. Surg. Case Rep..

[27] What Does It Mean to Have Inoperable Cancer? Dana-Farber. https://blog.dana-farber.org/insight/2017/12/inoperable-cancer-meaning/.

[28] Laylo J.C.V., Lim N.L., Remo J.J.V. (2021). Inflammatory myofibroblastic tumor of the urinary bladder: A prognostically favorable spindle cell neoplasm. Urol. Case Rep..

