# Supplementary material for: Rare Inflammatory Myofibroblastic Tumor of the Urinary Bladder: A Case Report and Review of the Literature
Source: J Clin Med. 2026 Mar 7;15(5):2047. doi: 10.3390/jcm15052047 (PMC12986090; doi:10.3390/jcm15052047)
Supplement: Supplementary file 1 [file jcm-15-02047-s001.zip › jcm-4161320-supplementary.pdf]

Supplementary table 1. Summary of Prior Case Reports of Inflammatory Myofibroblastic Tumor (IMT) of the Bladder

Study (Author, Year)	No. of patients	Age (yrs)	Sex	Presentation	Treatment	ALK Status	Outcome / Follow-Up
Scott et al., 1988	1	5	F	N/A	Radical cystectomy	N/A	N/A
	1	7	F	Hematuria	Pelvic exenteration	N/A	No recurrence (24 mo)
	1	5	F	Hematuria, dysuria	Partial cystectomy	N/A	No recurrence (18 mo)
	1	15	F	Hematuria	Partial cystectomy	N/A	No recurrence (24 mo)
	1	5	M	Hematuria	Local excision, chemotherapy, radiotherapy	N/A	No recurrence (18 mo)
	1	8	F	Hematuria	Local excision	N/A	No recurrence (19 mo)
	1	7	M	Strangury, dysuria	Local excision	N/A	N/A
	1	3	F	Hematuria	Radical cystectomy, hysterectomy	N/A	N/A
	1	12	F	Dysuria, palpable suprapubic mass	Local excision	N/A	No recurrence (74 mo)
	1	2	F	Dysuria, urinary retention	Local excision	N/A	No recurrence (29 mo)
Freud et al., 1991	1	2	M	Dysuria	Partial cystectomy	N/A	No recurrence (18 mo)
Lamovec et al., 1992	1	2	F	Dysuria	Radical cystectomy	N/A	No recurrence (8 mo)
	1	15	M	Hematuria	TURBT, partial cystectomy	N/A	No recurrence (12 mo)

	1	9	M	Hematuria, dysuria	TURBT, partial cystectomy	N/A	N/A
	1	8	F	Hematuria	Partial cystectomy	N/A	No recurrence (15 mo)
	1	10	M	Hematuria, dysuria	TURBT, partial cystectomy	N/A	N/A
	1	4	M	Hematuria	Partial cystectomy	N/A	No recurrence (24 mo)
	1	15	F	Hematuria	TURBT	N/A	N/A
	1	6	F	Hematuria	TURBT, partial cystectomy	N/A	No recurrence (7 mo)
	1	7	M	Hematuria	Partial cystectomy	N/A	No recurrence (2 weeks)
	1	7	F	Hematuria	TURBT	N/A	No recurrence (2 weeks)
	1	3	M	Hematuria	TURBT, partial cystectomy	N/A	No recurrence (42 mo)
Foschini et al., 1995	1	2	M	Hematuria	Local excision	N/A	N/A
	1	4	M	Hematuria	Local excision	N/A	No recurrence (96 mo)
Lakshmanan et al., 1997	1	4	M	Hematuria	Partial cystectomy	N/A	No recurrence (4 mo)
Netto et al., 1999	1	4	M	Dysuria, suprapubic pain	TURBT, partial cystectomy	N/A	No recurrence (10 mo)
	1	12	M	Hematuria, dysuria	TURBT, partial cystectomy	N/A	No recurrence (10 mo)
	1	7	F	Frequency, hematuria, dysuria, abdominal pain, palpable mass	TURBT	N/A	Small residual trigonal mass 3.5 years

Gardner et al., 1999	1	3	M	Lower abdominal pain, frequency, dysuria	Partial cystectomy	N/A	No recurrence (3 mo)
Meyer et al., 2000	1	3	M	Abdominal pain	Partial cystectomy	N/A	No recurrence (9 mo)
Choi et al., 2000	1	7	F	Frequency, pyuria, dysuria, flank pain, fever	TURBT → open excision	N/A	No recurrence (12 mo)
Iczowski et al., 2001	1	15	M	Hematuria, irritation	TURBT	N/A	No recurrence (22 mo)
Mergan et al., 2005	1	7	F	Hematuria	Partial cystectomy	ALK-	No recurrence (24 mo)
	1	15	M	Hematuria	Partial cystectomy	ALK-	No recurrence (60 mo)
	1	6	F	Hematuria	Partial cystectomy	ALK+	No recurrence (2 mo)
Flethcher et al., 2007	1	6	M	Lower abdominal pain, constipation, dysuria	Medications	N/A	No recurrence (22 mo)
Houben et al., 2007	1	10	F	Dysuria, frequency, sensation of complete voiding	Partial cystectomy	ALK+	No recurrence (6 mo)
Lecuona et al., 2012	1	3	M	Hematuria, lower urinary tract symptoms	Open excision	ALK+	No recurrence (7 mo)
Suer et al., 2012	1	10	F	Dysuria, enuresis	Open excision	ALK+	No recurrence (12 mo)
Chun et al., 2014	182	Mean age 38.9+ 16.6 years	51.7% F	Hematuria, dysuria, urinary frequency, lower	60.8% TURBT, followed by partial cystectomy (29.2%),	Most of the cases were ALK+	Five of 120 patients (4%) were noted to have a local tumor recurrence on

				abdominal pain, loin pain	radical cystectomy (9.2%) and cystoscopic biopsy (0.8%).		follow-up (mean follow-up was 30.0 mo +- 28.2 mo)
Alderman et al., 2014	1	38	F	Dysuria, pelvic pain	TURBT	ALK+	N/A
Collin et al., 2015	1	8	F	Hematuria	Open excision	ALK+	No recurrence (24 mo)
	1	7	F	Abdominal pain with micturition	Open excision	ALK+	No recurrence (24 mo)
Nkwam et al., 2016	1	62	F	Hematuria	TURBT, partial cystectomy	ALK+	No recurrence (6 mo)
Xu et al., 2018	1	25	M	Hematuria	TURBT	ALK+	No recurrence (48 mo)
	1	72	M	Hematuria	TURBT → partial cystectomy	ALK+	No recurrence (48 mo)
	1	33	F	Hematuria, urinary frequency	TURBT → partial cystectomy	ALK+	No recurrence (48 mo)
Libby et al., 2019	1	61	M	Hematuria + metastasis	TURBT + radical cystectomy	ALK–	Peritoneum and large intestines metastasis
Inamdar & Pulinthanathu, 2019	1	93	F	Hematuria	TURBT → progression	ALK –	Malignant transformation
Song et al., 2019	1	28	F	Severe hematuria	TURBT	ALK+	No recurrence (3 mo)
Li et al., 2020	8	Mean age 7.1 years	5 M<br>3 F	Lower urinary symptoms, hematuria, abdominal pain	Surgery	81.8% ALK+	No recurrence (43.3 mo)

Reinhart et al., 2020	1	43	F	Hematuria, dysuria	Crizotinib, partial cystectomy	ALK+	No recurrence (12 mo)
Matsui, 2021	1	55	M	Urinary retention	TURBT	ALK +	No recurrence (3 mo)
Laylo et al., 2021	1	28	F	Hematuria	TURBT, partial cystectomy	ALK+	No recurrence (18 mo)
Wang et al., 2021	1	74	M	Dysuria, hematuria	TURBT	ALK+	No recurrence (42 mo)
Balagobi et al., 2022	1	47	M	Dysuria, lower abdominal pain, urinary urgency and frequency	Partial cystectomy	ALK+	No recurrence (6 mo)
Marais et al., 2022	1	27	M	Hematuria, frequency	TURBT	ALK+	No recurrence (6 mo)
Chen et al., 2022	1	41	M	Hematuria, urgent urination	Partial cystectomy	In six of eight patients were ALK+	No recurrence (48 mo)
	1	22	M	Urgent urination, odynuria	Partial cystectomy		No recurrence (117 mo)
	1	73	M	Urgent urination, odynuria	Partial cystectomy		No recurrence (96 mo)
	1	62	F	Urgent urination	Partial cystectomy		No recurrence (100 mo)
	1	54	F	Hematuria	Partial cystectomy		No recurrence (47 mo)
	1	62	F	Hematuria	Radical cystectomy		No recurrence (48 mo)
	1	40	M	Hematuria	Partial cystectomy		No recurrence (54 mo)
	1	52	M	Hematuria	TURBT		No recurrence (24 mo)

	1	14	M	Hematuria, odynuria	Partial cystectomy		No recurrence (12 mo)
	1	16	M	Hematuria, odynuria	Partial cystectomy		No recurrence (48 mo)
	1	70	F	Dysuria	Partial cystectomy		No recurrence (3 mo)
	1	32	M	Urgent urination, odynuria	TURBT		No recurrence (6 mo)
	1	43	F	Hematuria, odynuria	Partial cystectomy		No recurrence (6 mo)
	1	45	M	Hematuria, odynuria	TURBT		No recurrence (6 mo)
Derimachkovski et al., 2023	1	59	M	Hematuria, dysuria, difficulty emptying the bladder	TURBT	ALK –	N/A

M – male

F – female

TURBT – transurethral resection of bladder tumor

ALK – anaplastic lymphoma kinase

N/A – not available

Mo – months
